# Electro- and Magneto-Modulated Ion Transport through Graphene Oxide Membranes

**DOI:** 10.1038/srep06798

**Published:** 2014-10-28

**Authors:** Pengzhan Sun, Feng Zheng, Kunlin Wang, Minlin Zhong, Dehai Wu, Hongwei Zhu

**Affiliations:** 1 School of Materials Science and Engineering, State Key Laboratory of New Ceramics and Fine Processing, Key Laboratory of Materials Processing Technology of MOE, Tsinghua University, Beijing 100084, China; 2 Department of Mechanical Engineering, Tsinghua University, Beijing 100084, China; 3 Center for Nano and Micro Mechanics, Tsinghua University, Beijing 100084, China

## Abstract

The control of ion trans-membrane transport through graphene oxide (GO) membranes is achieved by electric and magnetic fields. Electric field can either increase or decrease the ion transport through GO membranes depending on its direction, and magnetic field can enhance the ion penetration monotonically. When electric field is applied across GO membrane, excellent control of ion fluidic flows can be done. With the magnetic field, the effective anchoring of ions is demonstrated but the modulation of the ion flowing directions does not occur. The mechanism of the electro- and magneto-modulated ion trans-membrane transport is investigated, indicating that the electric fields dominate the ion migration process while the magnetic fields tune the structure of nanocapillaries within GO membranes. Results also show that the ion selectivity of GO membranes can be tuned with the electric fields while the transport of ions can be enhanced synchronously with the magnetic fields. These excellent properties make GO membranes promising in areas such as field-induced mass transport control and membrane separation.

Materials with nanopores and nanochannels have attracted significant research interest in recent years due to their potential applications in mass transport and nanofluidics^1^,^2^. Among them, carbon nanomaterials, *e.g.*, carbon nanotubes, which can afford an almost frictionless transport of water and effective ion exclusion due to their hydrophobic inner graphitic walls and narrow diameters, are believed to have potential applications in areas such as drug delivery, biomimetic selective ion transportation, water desalination and energy harvesting^3^,^4^,^5^,^6^,^7^,^8^,^9^,^10^. Recently, a growing number of attentions have been paid on graphene^11^, which has also been demonstrated as an excellent barrier film^12^,^13^. Typically, graphene is impermeable to all the gases and liquids despite its only one-atom thickness^14^. However, after introducing nanopores with various shapes, sizes and functional groups into the matrix, the nanoporous graphene can act as excellent sieving for various gases and ions in solutions, showing promises in molecular sieving^15^,^16^ and water desalination^17^. Graphene oxide (GO), prepared by the oxidization and exfoliation of graphite, is another promising 2D nanomaterial with unique structures, and is easy to synthesize and scale up^18^,^19^. GO sheets can be viewed as graphene attached with oxygen-containing functional groups, resulting in a 2D network of *sp*^*2*^*-* and *sp*^*3*^*-*bonded atoms^20^,^21^. After self-assembly, GO sheets are strongly held together through hydrogen bonds to form freestanding laminate with sufficient mechanical strength. The nanocapillaries, from the clustering of *sp*^*2*^ regions within the C-O matrix across all stacking layers, are responsible for the transport of water and ions through GO membranes^22^,^23^,^24^. Recently, it has been demonstrated that the anomalous unimpeded permeation of water through GO membranes can be achieved because of the formation of ice bilayer within the interlayer spaces and its melting transition at the edges of the flakes^22^,^25^. It has also been revealed that the coordination of transition metal ions with the *sp*^*3*^ matrix and the cation-π interactions of main group cations with the π clusters of GO sheets are responsible for the ion-selectivity of GO membranes^24^,^26^. All of these characteristics make GO membranes as promising candidates in many applications, such as water purification and ion separation.

In terms of ion trans-membrane transport through membranes with nanopores and nanochannels, the control of fluid flows is of crucial importance which can open new opportunities for water purification based on novel field-induced reverse osmosis^27^,^28^,^29^,^30^,^31^,^32^,^33^,^34^. Typically, there are two strategies to control the ion trans-membrane transportation: modulating the ion migration process in solutions and altering the structure of nanochannels. In order to modulate the ion migration process, an external electric field can be applied across the membrane. Previous experimental and theoretical results have also demonstrated that unusual magnetic properties of room-temperature ferromagnetism along with weak antiferromagnetism^35^,^36^,^37^,^38^,^39^,^40^,^41^,^42^,^43^ are present in defective graphene films, based on which the alteration of the structure of nanocapillaries may occur with a magnetic field across GO membranes during ion transport processes, just like the magnetic field-induced realignment of carbon nanotubes and graphene^44^,^45^.

In this work, the electro- and magneto-controlled ion trans-membrane transport properties of GO membranes are investigated based on the penetration of KCl, MgCl_2_, CaCl_2_ and FeCl_3_. After the ion transport processes, Auger electron spectroscopy (AES) is used to determine the concentration of residual ions within GO membranes. Additionally, the mechanism on the electro- and magneto-induced ion transport through GO membranes is discussed.

## Results

### Characterizations of GO membranes

**Figure 1:**
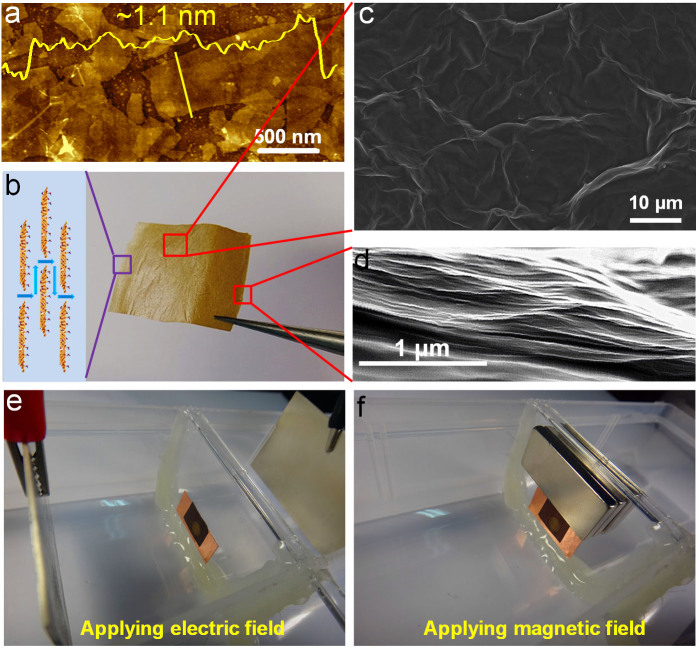
(a) AFM characterization of as-prepared GO sheets. (b) Photograph of the as-prepared free-standing GO membrane. (c,d) SEM characterizations of the GO membrane. (e,f) Experimental apparatus for ion transport through GO membranes when applying the electric and magnetic fields.

GO flakes were synthesized with the modified Hummers' method from potassium permanganate, sodium nitrite and concentrated sulfuric acid^18^,^19^. Figure 1a shows an AFM image of the as-synthesized GO sheets, which reveals that GO flakes have a lateral size of ~1 μm. The inset in Figure 1a demonstrates the monolayer nature of the as-prepared GO sheets. Freestanding GO membranes were prepared from the drop-casting of GO colloidal suspension (1.5 mg/mL, ~1 mL) onto a piece of smooth paper. After dried thoroughly, they were peeled off as shown in Figure 1b, which reveals the sufficient mechanical strength for freestanding manipulation (see Experimental Section and Figure S1). Figure S2 illustrates the cross-section of the as-prepared GO membrane, from which it can be evaluated that the as-prepared GO membranes possess a thickness of 1 ~ 2 μm. Figures 1c–d are SEM images of the surface and cross-section of GO membranes, showing that the as-prepared GO membranes have a wrinkled surface and a lamellar cross-section structure.

### Ion permeation experiments in the presence of both concentration gradients and external fields

The ion trans-membrane transport experiments were conducted on a self-made penetration apparatus (see Experimental Section, Figures 1e–f). Freestanding GO membranes were sealed with two-sided copper tape onto a plate with an aperture (~5 mm in diameter), which separated the source vessel from the drain vessel. 80 mL of certain salt solutions (0.1 mol/L) and deionized water were injected into the source and drain vessels, respectively. The conductivity variations of the drains were measured on a conductivity meter to reflect the penetrations of certain sources based on the fact that the conductivity is proportional to the concentration in diluted solutions. During the ion penetrations through GO membranes, electric and magnetic fields were applied as shown in Figures 1e and f, which enables us to investigate the electro- and magneto-induced ion trans-membrane transport properties of GO membranes.

**Figure 2:**
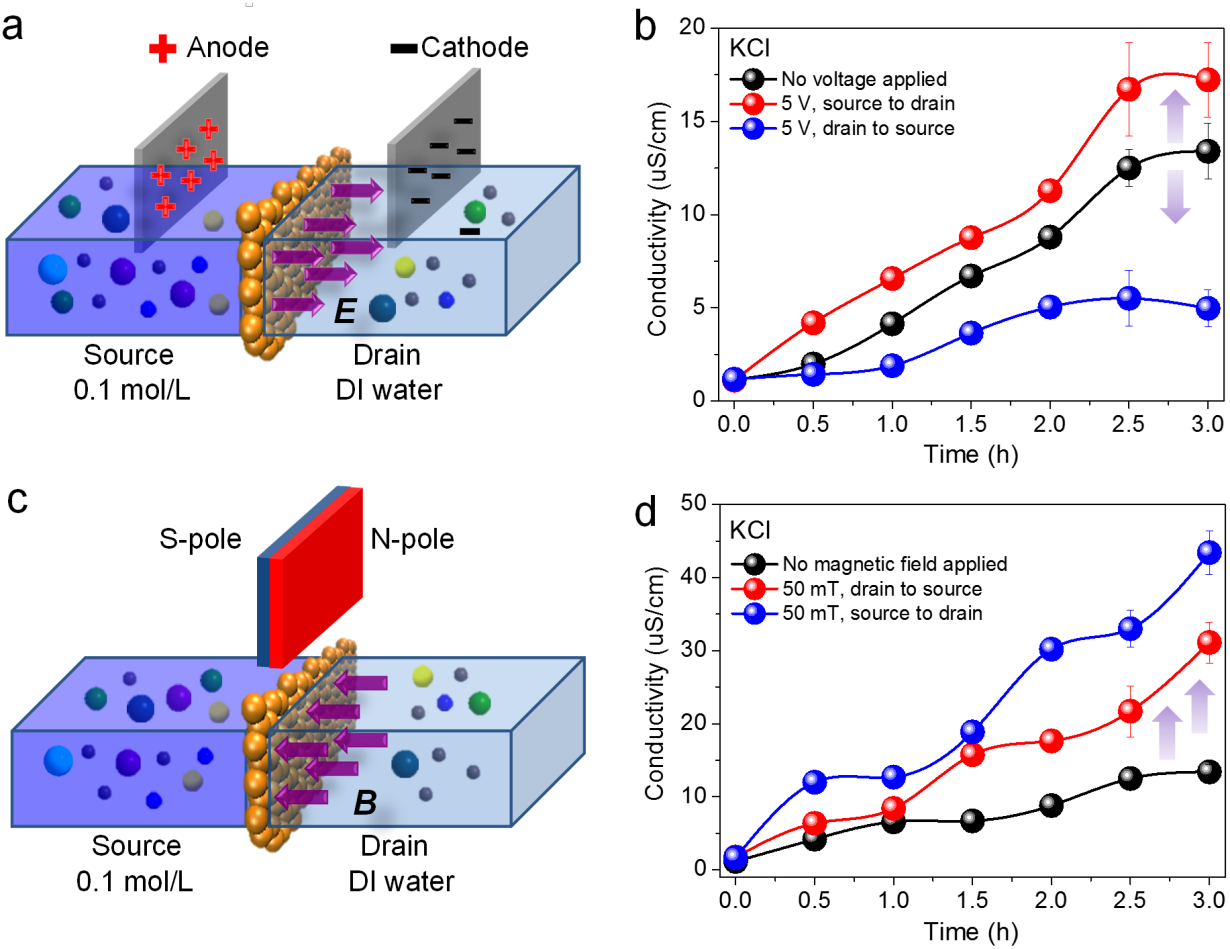
(a) Schematic diagram of ion transport through GO membranes when applying electric field. (b) The conductivity variations of the drains for KCl sources when applying forward and backward voltages. (c) Schematic diagram of ion transport through GO membranes when applying magnetic field. (d) The conductivity variations of the drains for KCl sources when applying magnetic fields with opposite directions. All of the experiments were repeated for 3 times and the data points were calculated by averaging all of the corresponding data from the same series of experiments. The error bars show the largest fluctuating ranges for the corresponding data points.

The electro-induced ion transport through GO membrane is illustrated in Figure 2a and the conductivity variations of the drains for KCl sources are plotted in Figure 2b. It is found that a forward electric field (source to drain) leads to the increment of penetration while the backward electric field (drain to source) decreases the penetration. In contrast, with a magnetic field (50 mT) on both directions across GO membranes as illustrated in Figure 2c, the penetrations of KCl are increased significantly as shown in Figure 2d. The same experiments have been done based on the penetrations of MgCl_2_ and CaCl_2_ sources, and similar results are obtained as shown in Figure S3. Additionally, the applied magnetic fields are further varied on the direction from drain to source. Conductivity variations of the drains for KCl, MgCl_2_ and CaCl_2_ are plotted in Figures S4a–c, which reveal that ion penetrations are increased gradually with the enhancement of magnetic field. These results demonstrate that the ion transport through GO membranes can be either increased or decreased with electric field, which is depended on its direction. However, the magnetic field can only enhance the ion penetrations through GO membranes.

### Ion permeation experiments in the presence of only external fields

**Figure 3:**
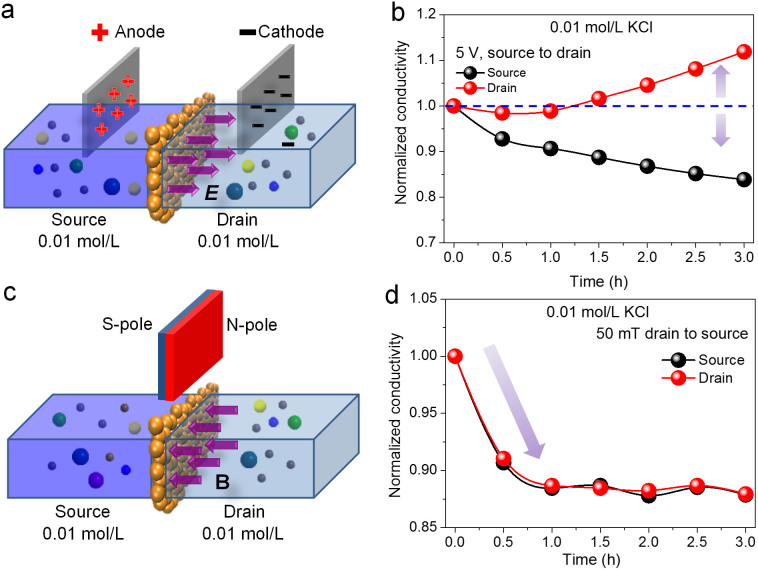
(a) Schematic drawing of the control of ion fluidic flows through GO membranes in the presence of electric fields. (b) The conductivity variations of the source and drain under the condition of applying 5 V voltage (source to drain) when the source and drain are both injected with 0.01 mol/L KCl. (c) Schematic drawing of the apparatus used to investigate the control of ion fluidic flows through GO membranes when applying magnetic fields. (d) The conductivity variations of the source and drain under the condition of applying 50 mT magnetic field (drain to source) when the source and drain are injected with 0.01 mol/L KCl solutions.

The control of ion fluidic flows through GO membranes within electric and magnetic fields is further investigated, as shown in Figure 3. 80 mL 0.01 mol/L KCl solutions were injected into the source and drain vessels at the same velocity to ensure that no concentration gradients were present between source and drain solutions. During the penetration, an electric field or a magnetic field was applied across the GO membrane (Figures 3a and c) and the conductivity variations of the sources and drains were measured. With an electric field across GO membrane (from source to drain) as illustrated in Figure 3a, obvious control of the ion flowing direction (from source to drain) occurs, which results in the increase of drain conductivity and the decrease of source conductivity (Figure 3b). If the voltage across GO membrane is reduced from 5 to 3 V (Figure S5), the modulation of the ion flowing direction is weakened gradually, indicating that the increase of electric field results in the enhancement of ion fluidic control. Interestingly, according to the conductivity variations in Figure 3b, it can be concluded that the decrease in source conductivity is larger than the increase of the drain when an electric field across GO membrane is applied. This asymmetrical change in conductivities might be attributed to the anchoring of ions around oppositely charged electrodes as well as around GO membranes. In contrast, with magnetic field across GO membrane (Figure 3d), conductivities of the source and drain are decreased significantly during the initial 1 h of penetration while maintain nearly unchanged afterwards. Curves of the conductivity variations in source and drain are nearly coincided together, indicating that no control of the ion fluidic directions occurs with the applied magnetic fields. Thus, it can be concluded that significant changes in ion transport through GO membranes occur with electric and magnetic fields. However, the electric fields cause effective modulation of ion flowing directions, while the magnetic fields cannot.

### Ion permeation experiments based on FeCl_3_ solutions and external magnetic fields

Specially, the magneto-modulated transportation of FeCl_3_ through GO membranes is investigated and the results are shown in Figures S6, 7. The reason why we chose to study the magneto-modulated permeance of FeCl_3_ is that the intense hydrolysis of Fe^3+^ would lead to the coexistence of abundant Fe^3+^ and H^+^ ions in solutions, which facilitates the investigation of trans-membrane permeation of transition metallic Fe^3+^ and H^+^ ions synchronously.

As shown in Figure S6, it reveals that when applying a magnetic field across the GO membrane with both directions, the penetrations of FeCl_3_ are enhanced (Figure S6a). Furthermore, increasing the strength of the magnetic field applied (from 25 to 50 mT with the direction of drain to source) leads to the gradual increase of penetration through GO membranes (Figure S6b). The insets in each figure show the accurate concentrations of Fe^3+^ in drains after penetration for 3 h carried out by atomic emission spectroscopy, revealing that the trans-membrane transport of Fe^3+^ can be enhanced significantly by the magnetic fields applied. These results are similar to the cases of KCl, MgCl_2_ and CaCl_2_, as shown in Figures 2, S3 and S4.

In addition to conductivity measurements, the pH values of the drains were also recorded during the application of magnetic fields, as shown in Figures S6c–d. Surprisingly, it reveals that the trans-membrane transport of protons is not affected by the magnetic field applied. The results in Figure S6 further imply that the transportations of metallic cations and H^+^ ions through GO membranes should follow rather different mechanisms, which enables the effective separation of protons from Fe^3+^ cations during the trans-membrane permeation of iron-based electrolytes.

In order to investigate the different magnetic effects on the transport of Fe^3+^ and H^+^ ions through GO membranes, the experiments of modulating the ion fluidic flows were done based on FeCl_3_ solutions and a 50 mT magnetic field, as illustrated in Figure 3c. During the penetration, the conductivity and pH variations of the source and drain were both measured with time, as shown in Figure S7. It reveals that the conductivities of the source and drain are both decreased with penetration while the pH values are not changed under the condition of magnetic field applied. As the penetration of protons is not affected when applying a magnetic field across the GO membrane (Figure S7b), it can be concluded that the decrease of conductivities in source and drain (Figure S7a) is attributed to the magnetic-modulated trans-membrane transport of Fe^3+^.

### Control experiments with cellulose microfilters and AES characterizations on GO membranes

To further reveal the tuning mechanism of electric and magnetic fields on the ion trans-membrane transport through GO membranes, control experiments were conducted with commercial cellulose microfilters (~200 nm in aperture, ~80% in porosity, as shown in Figure S8). With 5 V voltage along the direction from source to drain across the microfilter (Figure S8a), the conductivity variations of source and drain show similar change tendencies as in the cases of GO membranes, which indicates that electric fields play a major role in tuning the ion migration. However, the effect of modulating the ion flowing directions through GO membrane is greater than that through microfilter, which is presumably attributed to the diverse interactions of cations and anions within GO membranes. In detail, in aqueous environment, the ionization of oxygen functionalities decorated on GO should charge the GO membranes negatively. Besides the external electric fields, the electrostatic attractions and the cation-π interactions^26^ between the cations and GO membranes can act as extra driving forces for the modulation of ion fluidic flows through GO membranes, which are absent in the typical polymer-based microfilters, further leading to the greater effect of modulating ion flowing directions in the case of GO membranes than microfilters. In contrast, when a 50 mT magnetic field is applied along the direction from drain to source across the microfilter (Figure S8c), the conductivities of the source and drain are not changed markedly compared to that from GO membrane (Figure S8d), demonstrating that the magnetic field is ineffective to dominate the ion migration behavior in solutions but it might play an important role in altering the internal structure of GO membranes. The differing effects of electric and magnetic fields on the trans-membrane transport of ions through GO membranes can also be found from the AES characterizations of GO membranes after ion penetrations, as shown in Figure S9. When an electric field is applied across the GO membrane, the concentration of residual cations within GO membranes is increased significantly (Figure S9b), indicating that the forward electric field enhances the ion flow through GO membrane to force more ions to interact with the GO nanocapillaries, which results in a larger amount of cations remained within GO membranes. However, in the case of magnetic field, the concentration of cations remained within GO membrane is reduced slightly (Figure S9c). Because the magnetic field across the GO membrane causes significant changes in ion penetration (Figures 2 and 3) while it has negligible effects on ion migration in solutions (Figure S8), it can be deduced that magnetic fields can alter the internal stacking structure of GO membranes to result in an enhanced transport of ions, while the electric fields only dominate the ion migration process in solutions and have negligible effects on GO membrane.

## Discussion

### Mechanism

**Figure 4:**
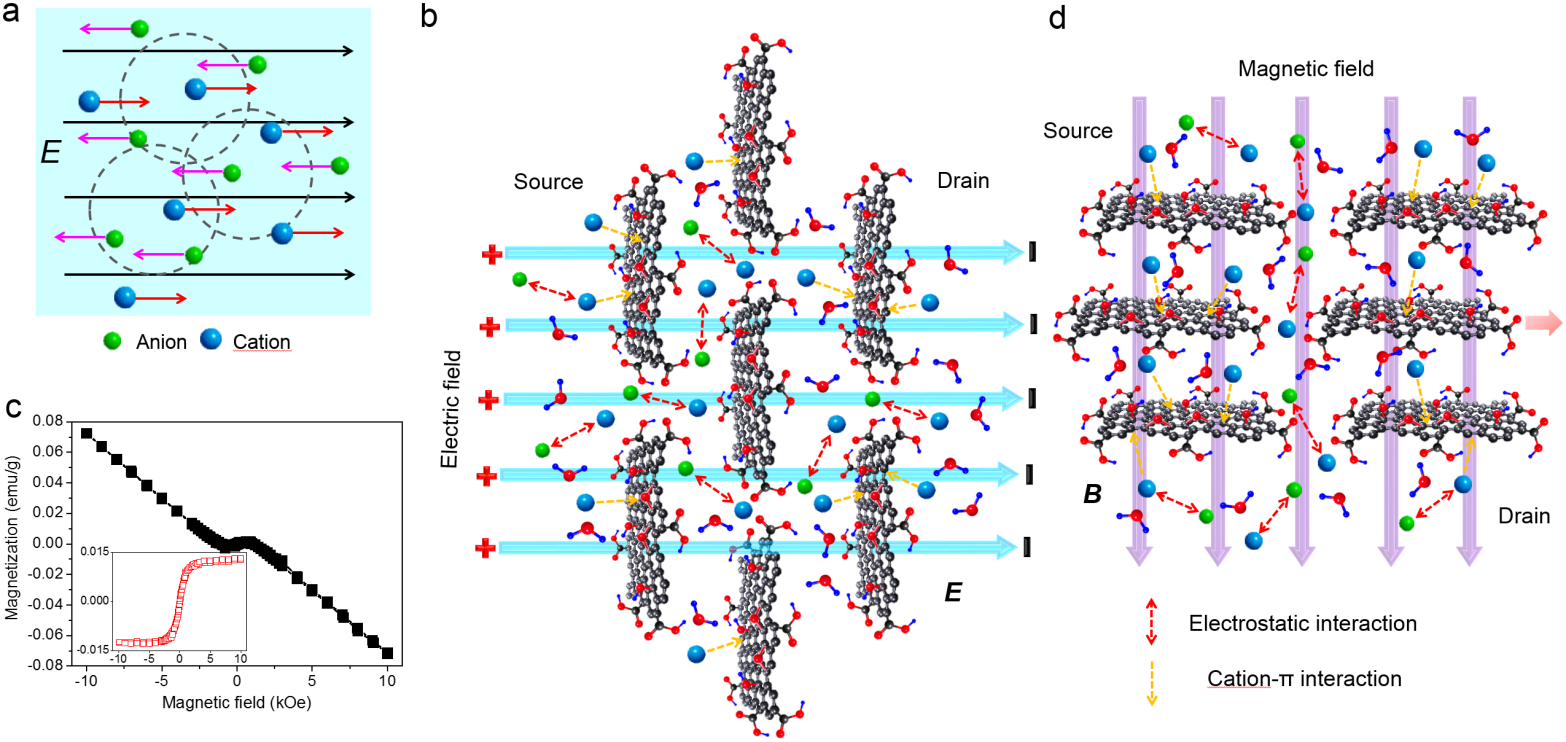
(a) Diagrammatic sketch for the formation and motions of the ionic clouds under electric fields. (b) Schematic diagram of the mechanism proposed for the electro-induced ion trans-membrane transport through GO membrane. (c) *M-H* curves of the GO membrane used in the experiments. The inset shows the excess moment Δ*M* as a function of *H* of GO membrane after subtracting the diamagnetic background, showing the ferromagnetic component. (d) Schematic diagram of the mechanism proposed for the magneto-induced ion trans-membrane transport through GO membrane.

The mechanism on the ion trans-membrane transport through GO membranes with external electric and magnetic fields is discussed in the following. In a typical aqueous solution, the hydrated ions are rearranged to reach an equilibrium state with two opposite forces; the electrostatic interaction leads to the formation of a partially ordered ionic cloud in which each ion (central ion) is surrounded by several oppositely charged ions; on the other hand, the random thermal jigging of ions disturbs the partially ordered structure of ionic cloud (Figure 4a). When an external electric field is applied, cations and anions will be accelerated in opposite directions and the symmetry of ionic cloud will be damaged (Figure 4a). Due to the opposite migrations of ionic clouds and central ions, the asymmetrical ionic clouds will hinder the transport of central ions and the central ions tend to attract the ionic clouds in the contrary direction. The electric force *F* can be calculated as the following: 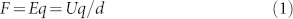
where *U* is the voltage applied across GO membranes, *d* is the distance between two planar electrodes and *q* is the charge of ion.

In this study, *U* is 5 V, *d* is ~10 cm and the electric force *F* of monovalent ions can be calculated as 8.0 × 10^−18^ N. On the other hand, the radius of ionic cloud can be expressed as the Debye screening length *κ*^−1^ and the electrostatic interaction *F'* between monovalent cations and anions can be calculated according to the following equation, 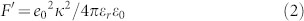
where *e*_0_ is the unit charge; *κ*^2^ = 2*e*_0_^2^*N*_A_*ρ*_s_*m*^0^***I***/*ε_r_ε_0_k*_B_*T*, where *N*_A_ is the Avogadro's number, *ρ*_s_ is the density of solvent, *m*_0_ = 1 mol/kg, ***I*** is the ionic strength, *ε_r_* is the relative permittivity, *ε_0_* is the vacuum dielectric constant, *k*_B_ is the Boltzmann constant and *T* is the temperature.

At 25°C, the electrostatic interaction *F'* between monovalent cations and anions in aqueous solutions (*ε_r_* = 78.54, *ρ*_s_ = 997 kg/m^3^) can be evaluated as ~3.18 × 10^−12^ N, which is six orders of magnitude greater than the electric force *F* with external electric fields. Therefore, the central ions cannot be separated from their ionic clouds and only the relative ion transport rates and directions can be adjusted with the external electric fields. In terms of ion trans-membrane transport, due to the ionization of oxygen-containing functional groups in aqueous environment, the GO membranes would be negatively charged, resulting in the attraction of cations and repulsion of anions. The cations would firstly migrate into and through GO membranes and interact with the negatively charged GO nanocapillaries, while the anions would be attracted electrostatically by the cations into the drains as shown in Figure 4b. Therefore, the forward electric fields can accelerate the migration of cations and the backward electric fields hinder the transport of cations, while the anions would always be dragged passively by the electrostatic attractions from the cations to penetrate through GO membranes. Typically, the ion transport in electrolyte solutions and across GO membranes can be described by the extended Nernst-Planck equations: 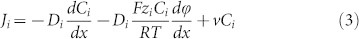
In this equation, *D_i_* is the diffusion coefficient, *F* is the Faraday constant, *φ* is the electric potential and *v* is the linear convective velocity along the direction *x*. The three terms in Eq. 3 represent three mass transportation modes across GO membranes: (1) diffusion driven by concentration gradients; (2) diffusion by electric potential gradients; (3) convection by pressure gradients. This equation cannot be solved directly, but it can be used for explaining the modulation of ion transport through GO membranes in the presence of both concentration gradients and external electric fields qualitatively. In detail, in the presence of both a forward concentration gradient and a forward electric field across the GO membrane (the terms 1 and 2 in Eq. 3 describing the diffusions driven by concentration gradient and electric potential gradient possess the same sign), the diffusion driving force and the electric force should reinforce each other to result in an enhanced ion transport through GO membranes. On the other hand, in the presence of a forward concentration gradient and a backward electric field across the GO membrane (the terms 1 and 2 in Eq. 3 describing the diffusions driven by concentration gradient and electric potential gradient possess opposite signs), the diffusion driving force and the electric force should work against each other to result in a weakened ion transport through GO membranes, as demonstrated in Figure 2b. When the source and drain are both filled with 0.01 mol/L electrolyte solutions and an electric field is applied across the GO membranes, only the electric driving force is present across the GO membrane and the electro-induced ion transport would be controlled by the inherent properties of ions (*e.g.* mobilities *u*_i_, charges, sizes and masses). With the radius of ions in Table S1, the ion mobility *u*_i_ can be calculated as the following: 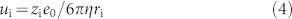
where *z*_i_*e*_0_ is the charge of ion, *η* is the viscosity of solvent and *r*_i_ is radius of the hydrated ion.

The external electric forces and the electrostatic attractions from GO membranes would drive the transport of cations from source to drain against the electrostatic hindrance from the anions, while the anions would be dragged by the cations to penetrate through GO membranes passively against the opposite external forces. After the cations and anions penetrate through the GO membranes and reach the drains, the migration of ions would be influenced by their inherent characters. In KCl solutions, due to the same monovalent charges and similar sizes of the hydrated K^+^ and Cl^−^ (Table S1), the mobilities of hydrated K^+^ and Cl^−^ are comparable (Eq. 4) and the external electric forces (Eq. 1) are equal in opposite directions, which result in the slow diffusion of K^+^ and Cl^−^ in drains and the delayed aggregation of ions around the cathode, and further the obvious control of ion fluidic flows for KCl solutions (Figure 3b). When GO membranes are changed to microfilters (Figures S8a and b), the relative changes of ion concentrations in the source and drain are much smaller, which can be attributed to the lack of electrostatic interaction between the ions and microfilters.

Previous experimental and theoretical studies have demonstrated the existence of prominent ferromagnetic along with antiferromagnetic features in most of graphene samples, presumably due to the presence of sheet defects and edge states^36^,^37^,^38^,^39^,^40^,^41^,^42^. The magnetic properties of GO membranes, which are composed of stacked and overlapped monolayer GO sheets, are also investigated in this study as shown in Figure 4c. Obviously, a dominant diamagnetism is evident in our GO samples at 300 K (demonstrated by the linear relation with inverse proportion between mass magnetization *M* and *H*). After subtracting the diamagnetic background, as shown in the inset of Figure 4c, it reveals that a weak room-temperature ferromagnetism is coupled in the GO membranes (demonstrated by the hysteresis characteristic^46^ between excess moment Δ*M* and *H* shown in the inset of Figure 4c). Therefore, based on the magnetic properties of GO membranes shown in Figure 4c, we conclude that the dominant diamagnetic accompanied with weak ferromagnetic behavior is present in GO membranes, which is in contrary to most of previous studies^36^,^37^,^40^,^41^,^42^. Presumably, the emergence of dominant diamagnetism is due to the inherent structural defects in graphene crystals^46^, while the room-temperature ferromagnetism is attributed to the external introduction of various defects such as adatoms, point defects, functionalities and magnetic edge states^35^,^36^,^37^,^38^,^39^,^40^,^41^,^42^,^43^,^47^. When a magnetic field across GO membrane is applied, due to the presence of room-temperature ferromagnetism in our GO sample, as shown in the inset of Figure 4c, the GO sheets within the membrane would be stacked more orderly^44^,^45^, as illustrated in Figure 4d. The more ordered stacking of GO sheets can shorten the length of GO nanocapillaries, which should facilitate the smooth trans-membrane transport of ions within the GO membrane (Figure 4d). Therefore, the magnetic field would lead to the enhancement of ion penetration through GO membranes (Figures 2, S3 and S4). During the magneto-response process, the GO flakes can be considered as numerous weak ferromagnetic bodies and the flake realignment process is independent of the direction of the magnetic field applied, further leading to the phenomenon that the magneto-modulated ion trans-membrane permeation is independent of the field direction (Figures 2, S3 and S6). Additionally, the shortening of GO nanocapillaries would also reduce the concentrations of cations remained within the membranes, which is in agreement with the AES results in Figure S9c. When 0.01 mol/L certain electrolytes are injected into both the source and drain (Figure 3c), due to the strong attractions of metallic cations and the electrostatic repulsions of anions with the negatively charged GO membranes^24^,^26^, ordered double electric layers would be formed on the GO interfaces and the ions would be anchored tightly around the GO membranes, which might result in the decrease of ion concentrations in the main source and drain (Figure 3d). In contrast, for the transport of protons through GO membranes, the rapid migration of protons relies on the fast propagation through the hydrogen-bonding networks along the water layers formed in between the GO flakes^48^, which shouldn't be affected seriously by the more ordered stacking of GO sheets. Therefore, the permeation of protons is not changed by the magnetic field applied, as shown in Figures S6c and d. In the case of ion trans-membrane transport through microfilters, the changes of ion concentrations in the source and drain are not obvious, compared to GO membranes, presumably due to the absence of strong interactions between ions and microfilters (Figures S8d).

### Electro- and magneto-modulated trans-membrane transport of hybrid sources

**Figure 5:**
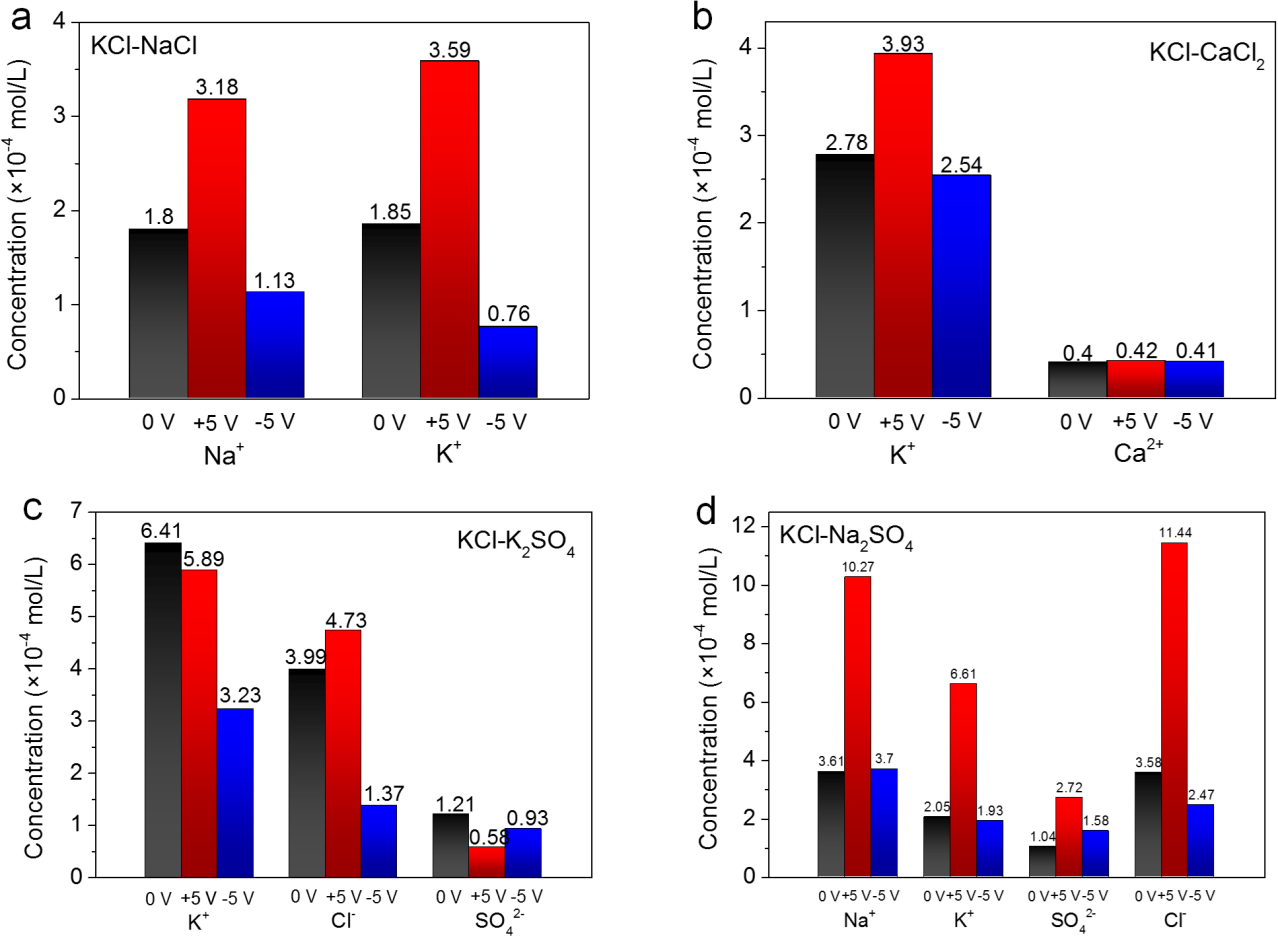
Ion concentrations of the drains during the penetrations of (a) KCl-NaCl, (b) KCl-CaCl_2_, (c) KCl-K_2_SO_4_ and (d) KCl-Na_2_SO_4_ hybrid sources when applying electric fields with opposite directions.

**Figure 6:**
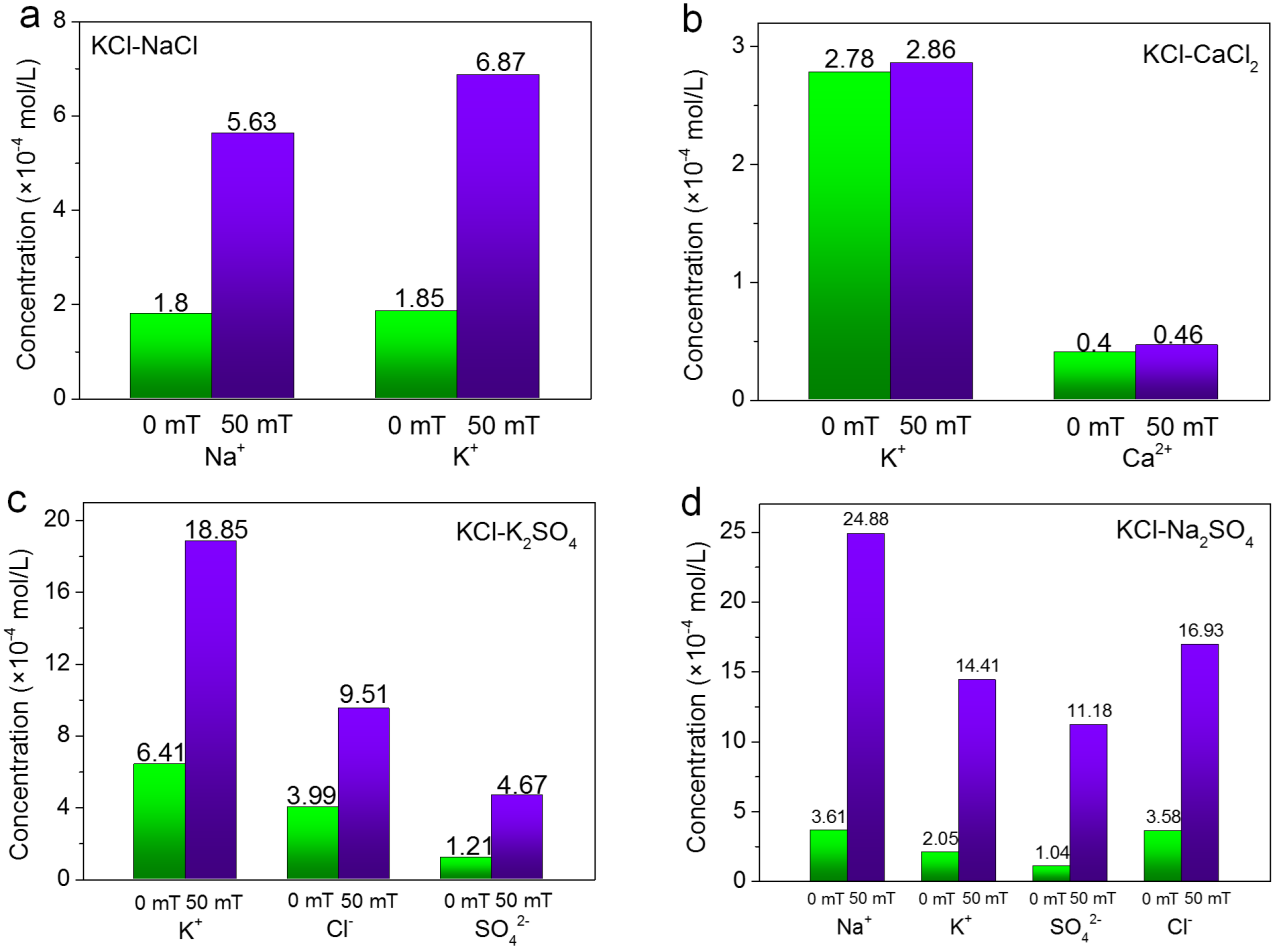
Ion concentrations of the drains during the penetrations of (a) KCl-NaCl, (b) KCl-CaCl_2_, (c) KCl-K_2_SO_4_ and (d) KCl-Na_2_SO_4_ hybrid sources when applying magnetic fields (drain to source).

Furthermore, the transport properties of hybrid sources through GO membranes under electric and magnetic fields are studied (illustrated in Figures 2a and c). The concentrations of ions in drains after 3 h of penetration were quantified with atomic emission spectroscopy and liquid chromatography as shown in Figures 5 and 6. With electric fields across GO membranes, it is found in Figure 5 that, for most hybrid sources except KCl-K_2_SO_4_, the forward electric field increases the ion penetration while the backward electric field decreases the penetration. Exceptionally for KCl-K_2_SO_4_, the forward electric field weakens the ion transport through GO membranes (Figure 5c), presumably due to the fact that the higher negatively charged SO_4_^2−^ would experience greater drags in the presence of forward electric fields and the penetrations of SO_4_^2−^ would be hindered markedly. On the other hand, the strong attraction between SO_4_^2−^ and K^+^ ions would also slow the trans-membrane transport of K^+^ significantly, as demonstrated in Figure 5c. Notably, the selectivity of GO membranes towards different ions can be tuned with the electric fields on different directions. For example, in the case of KCl-NaCl (Figure 5a), the penetration of K^+^ is preferred with forward electric fields while Na^+^ is more favored with backward electric fields. For KCl-CaCl_2_ (Figure 5b), the penetration of K^+^ can be tuned significantly while the penetration of Ca^2+^ is not affected by the electric fields applied. In the cases of KCl-K_2_SO_4_ and KCl-Na_2_SO_4_ hybrid sources (Figures 5c and d), the penetrations of anions through GO membranes are strongly influenced by the electric fields. The forward electric field favors the transport of Cl^−^ while the ratio of SO_4_^2−^ ions is enhanced in the presence of backward electric field. In contrast, when magnetic fields are applied across GO membranes, the penetrations of cations and anions are enhanced synchronously and the selectivity towards different ions is not changed significantly as shown in Figure 6.

## Conclusion

In summary, the control of ion trans-membrane transport through GO membranes has been achieved by electric and magnetic fields. It is found that the electric field can either increase or decrease the transport of ions depending on different directions, while the magnetic field can enhance the ion penetration through GO membranes only. Significant control of ion fluidic flows occurs when electric fields are applied across GO membranes while that does not occur with magnetic fields. Furthermore, mechanism of the electro- and magneto-induced ion trans-membrane transport is investigated, demonstrating that the electric fields dominate the ion migration in solutions while the magnetic fields alter the structure of nanocapillaries in GO membranes. Finally, the transport properties of hybrid sources through GO membranes with electric and magnetic fields are studied, revealing that the electric fields can adjust the selectivity of GO membrane towards different ions while the magnetic fields can enhance the ion transport synchronously. These excellent properties of GO membranes make them promising in areas such as field-induced mass transport control and membrane separation.

## Experimental Section

### GO membrane preparation

The monolayer GO flakes were prepared by the modified Hummers' method from potassium permanganate, sodium nitrite and concentrated sulfuric acid according to the previous methods^18^,^19^. After that, the as-prepared GO flakes were dispersed in deionized water by sonication to form the 1.5 mg/mL GO colloidal solutions. Then we took a piece of label paper (shown in Figure S1a) and peeled off all the labels. The remaining smooth paper underneath was finally utilized for the subsequent GO membrane formation, as shown in Figure S1b. Several droplets of GO solutions (~1 mL) were drop-casted onto this smooth paper, as illustrated in Figure S1c. After that, the as-drop-casted GO droplets were left to dry spontaneously in air atmosphere, followed by detaching off from the smooth paper to form the free-standing GO membranes, as shown in Figure S1d.

### Penetration experiments

The ion trans-membrane transport experiments were conducted with a home-made penetration apparatus (Figures 1e and f). The penetration apparatus was composed of two vessels (source and drain) separated by a plastic plate. An aperture with a diameter of 5 mm was located in the center of the plastic plate. A piece of free-standing GO membrane was sealed with double-sided copper tape (introducing a hole with the same diameter of 5 mm in the center) onto the aperture on the plastic plate so that the solutions in sources and drains could be directly connected by GO membranes to facilitate ion trans-membrane transport without passing through any bulky supports. The electric and magnetic fields were applied as illustrated in Figures 1e and f. For the application of electric fields, a pair of silver electrodes (99.99% in purity) was fixed across the GO membrane with a distance of ~10 cm and voltages were applied between the silver electrodes (Figure 1e). For the application of magnetic fields, several pairs of magnets (Nd-Fe-B permanent magnetic alloy) were fixed above the GO membrane (Figure 1f) and the magnetic fields located at the GO membrane were measured by a magnetometer (TES, WT10A). During the penetration experiments, 80 mL of 0.1 mol/L certain electrolytes and deionized water were injected into the source and drain vessels respectively and the conductivity variations of the drains were measured with time under mild mechanical stirring on a conductivity meter (INESA, DDS-307) to investigate the coexistence of concentration gradients and external fields on the ion transport behavior through GO membranes (Note: In the case of electric fields applied, the conductivity variations of the drains were measured after the voltages were tuned off to eliminate the effect of external electric fields on the conductivity measurement. After that, the voltages were tuned on again). In terms of the investigation on the control of ion fluidic flows through GO membranes under the applications of electric and magnetic fields, 80 mL 0.01 mol/L certain electrolytes were injected into both the source and drain vessels with the same speed and the conductivity variations of both the sources and drains were measured with time under stirring. In the case of magneto-modulated transportations of FeCl_3_ sources through GO membranes, the pH values of the sources and drains were also measured with time on a pH meter (SANXIN, MP523) to study the penetration behavior of protons. After the penetration experiments, a simple hydrostatic pressure experiment was done to ensure the stability of the GO membranes in solutions. Briefly, one vessel of the penetration apparatus was filled with water while the other one was left empty. If no water flowed across the GO membrane during the 30 min of testing, it was believed that the GO membrane kept intact and stable during the penetration experiments.

### Characterizations

The as-prepared GO sheets and GO membranes were characterized by atomic force microscopy (AFM, Agilent 5100), scanning electron microscope (SEM, LEO 1530, 10kV) and Auger electron spectroscopy (AES, PHI 700). The magnetism of GO membranes was measured by SQUID magnetometer (Quantum Design MPMS–XL, USA). The concentrations of certain cations and anions in drains after 3 h of penetration were carried out by atomic emission spectroscopy (IRIS Intrepid II) and liquid chromatography (SY-5000), respectively.

## Author Contributions

H.W.Z. and P.Z.S. conceived and designed the experiments. P.Z.S, F.Z. performed the experiments. M.L.Z., K.L.W. and D.H.W. conducted the theoretical analysis. P.Z.S. and H.W.Z. co-wrote the manuscript.

## Supplementary Material

Supplementary Information SUPPLEMENTARY INFO
