# Supplementary material for: Electro- and Magneto-Modulated Ion Transport through Graphene Oxide Membranes
Source: Sci Rep. 2014 Oct 28;4:6798. doi: 10.1038/srep06798 (PMC4210866; doi:10.1038/srep06798)
Supplement: Supplementary Information — SUPPLEMENTARY INFO [file srep06798-s1.pdf]

Supplementary Information

Electro- and Magneto-Modulated Ion Transport through Graphene Oxide Membranes

Pengzhan Sun[1], Feng Zheng[1], Kunlin Wang[1], Minlin Zhong[1], Dehai Wu[2], Hongwei Zhu[1,3*]

[1]School of Materials Science and Engineering, State Key Laboratory of New Ceramics and Fine Processing, Key Laboratory of Materials Processing Technology of MOE, Tsinghua University, Beijing 100084, China

[2]Department of Mechanical Engineering, Tsinghua University, Beijing 100084, China

[3]Center for Nano and Micro Mechanics, Tsinghua University, Beijing 100084, China

*Corresponding author. Email: hongweizhu@tsinghua.edu.cn.

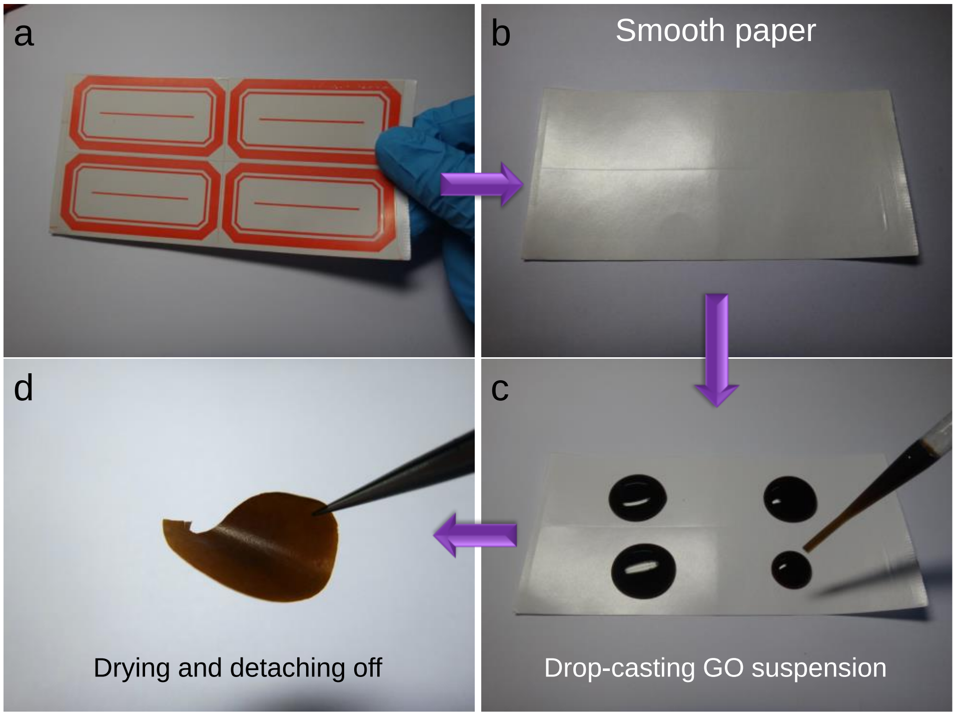

Figure S1. Free-standing GO membrane preparation procedures. (a) Photograph of a piece of label paper used for the formation of smooth paper. (b) Photograph of the smooth paper used for drop-casting GO solutions to form the GO membranes. (c) Photograph of the processes for drop-casting GO solutions (1.5 mg/mL). (d) Photograph of a piece of free-standing GO membrane formed after drying and detaching off from the smooth paper.

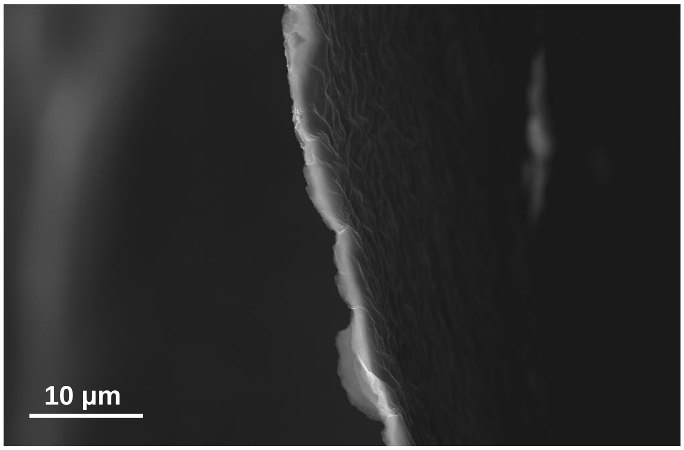

Figure S2. Cross-section SEM image of the as-prepared GO membrane, from which the thickness of the GO membranes can be evaluated as around 1~2 μm.

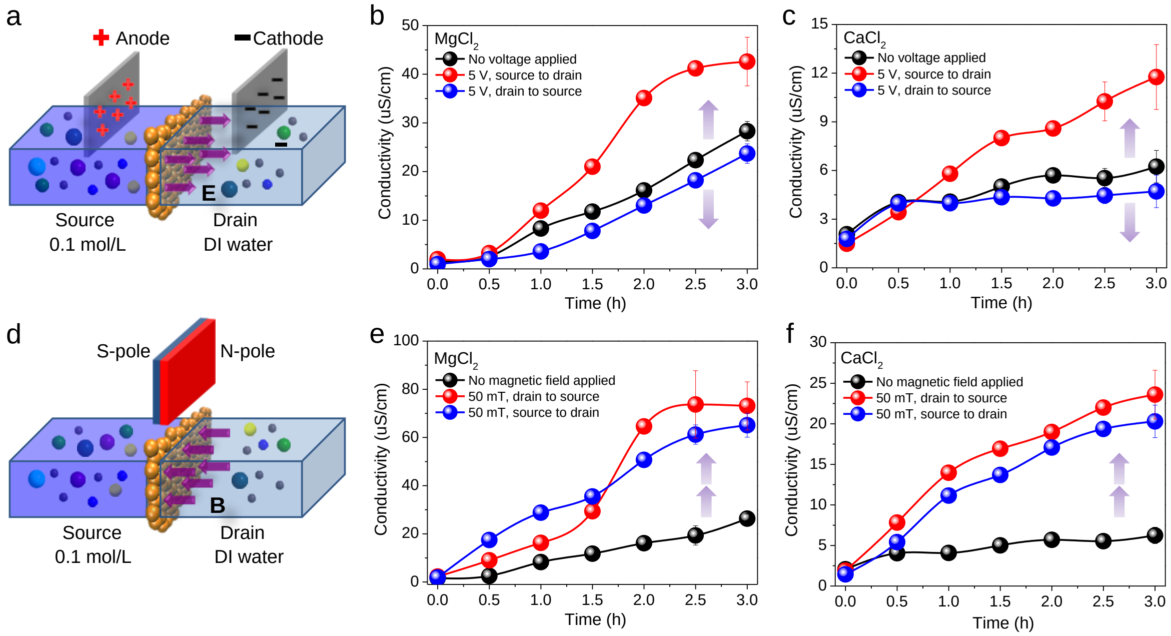

Figure S3. (a) Schematic diagram of ion transport through GO membranes when applying electric field. The conductivity variations of the drains for (b) $MgCl_2$ and (c) $CaCl_2$ sources when applying forward and backward voltages. (d) Schematic diagram of ion transport through GO membranes when applying magnetic field. The conductivity variations of the drains for (e) $MgCl_2$ and (f) $CaCl_2$ sources when applying magnetic fields with opposite directions. All of the

experiments were repeated for 3 times and the data points were calculated by averaging all of the corresponding data from the same series of experiments. The error bars show the largest fluctuating ranges for the corresponding data points.

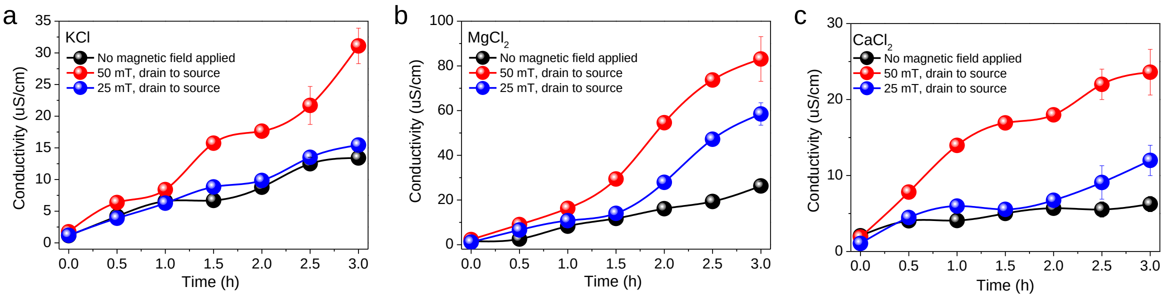

Figure S4. Drain conductivity variations of (a) KCl, (b) $MgCl_2$ and (c) $CaCl_2$ when applying magnetic fields with increasing values (drain to source). All of the experiments were repeated for 3 times and the data points were calculated by averaging all of the corresponding data from the same series of experiments. The error bars show the largest fluctuating ranges for the corresponding data points.

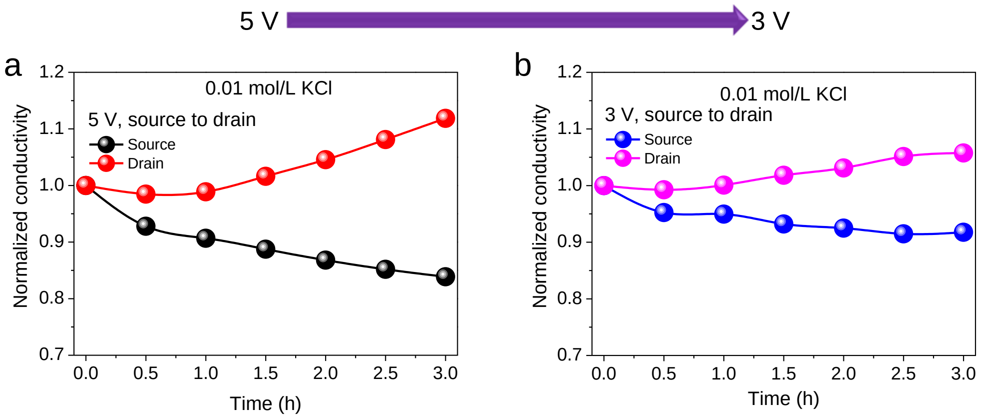

Figure S5. Conductivity variations of the source and drain under (a) 5 V (source to drain) and (b) 3 V when the source and drain are both filled with 0.01 mol/L KCl solutions.

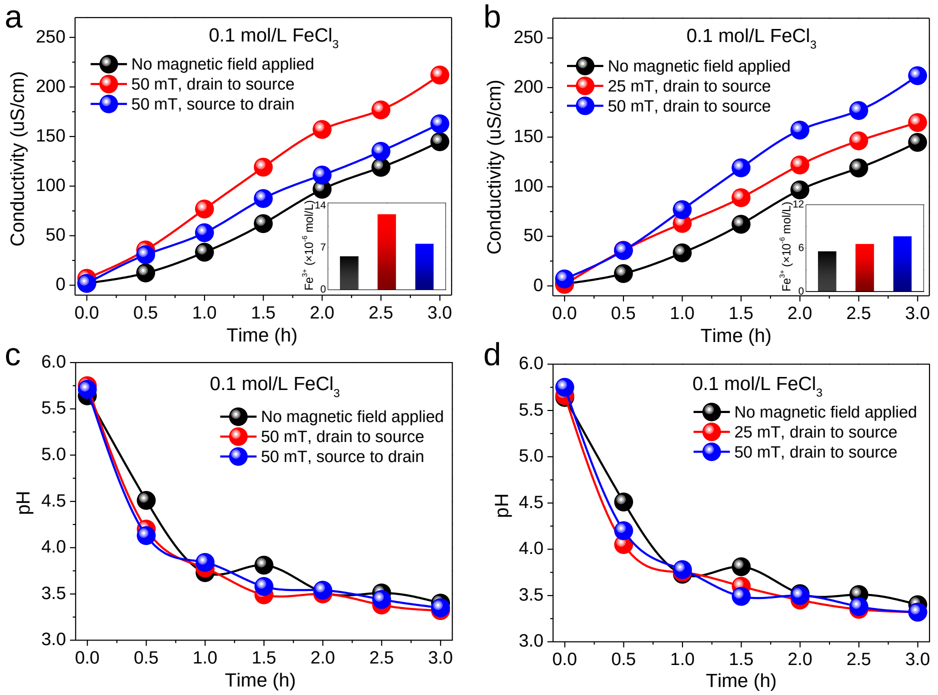

Figure S6. Conductivity and pH measurements of the drains for the penetration of 0.1 mol/L $FeCl_3$ sources through GO membranes under the application of magnetic fields with different directions (a,c) and values (b,d). The insets in a and b are the accurate concentrations of Fe^{3+} in drains after penetration for 3 h carried out by atomic emission spectroscopy.

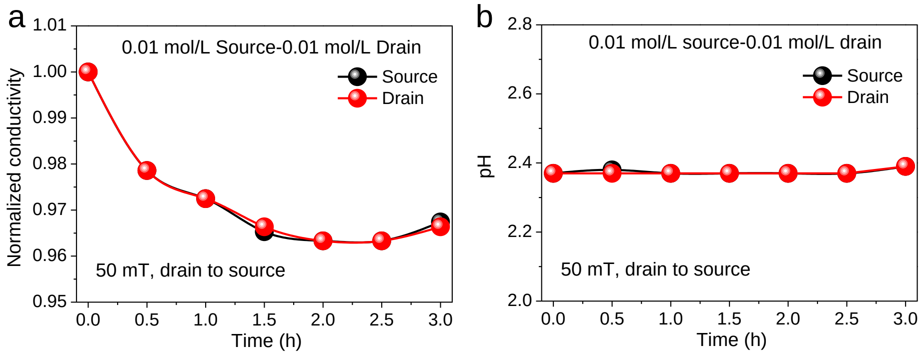

Figure S7. Conductivity (a) and pH (b) variations of source and drain under the application of a 50 mT magnetic field with the direction of drain to source when the source and drain vessels are both filled with 0.01 mol/L $FeCl_3$ solutions, respectively.

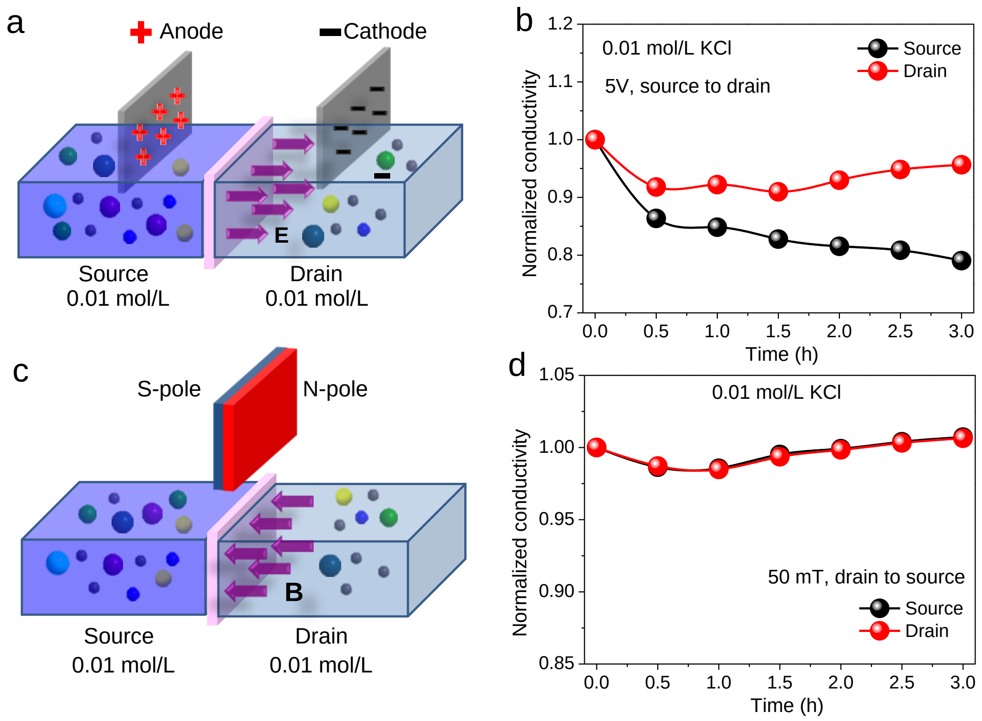

Figure S8. Control experiments with commercial cellulose microfilters (220 nm in aperture, ~80% in porosity). (a) Schematic for the control of ion fluidic flows through microfilters when applying electric fields. (b) Conductivity variations of the source and drain under 5 V (source to drain) when the source and drain are both filled with 0.01 mol/L KCl solutions. (c) Schematic for the case of applying magnetic fields. (d) Conductivity variations of the source and drain under a 50 mT magnetic field (drain to source) when the source and drain are both filled with 0.01 mol/L KCl solutions.

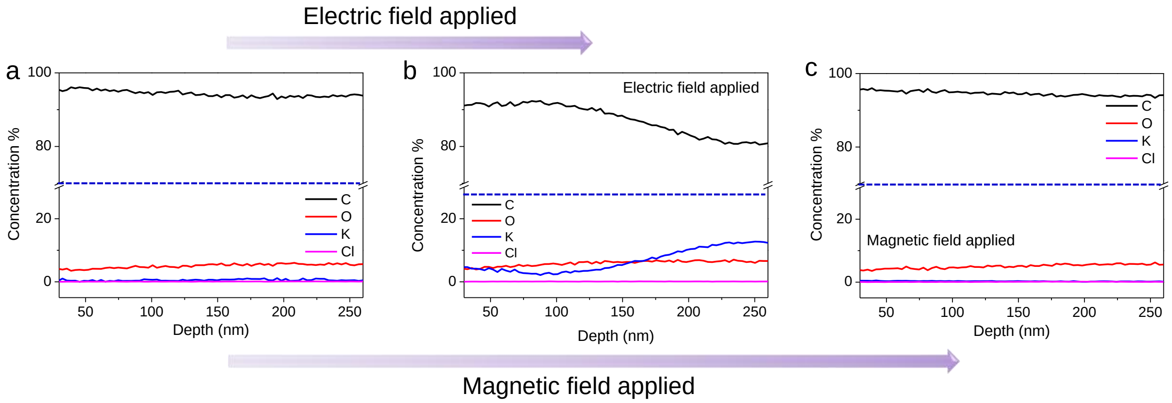

Figure S9. AES characterizations (ion distributions) of GO membranes after the penetrations of KCl sources under 5 V and 50 mT. (a) AES characterization of GO membrane after the penetration of KCl sources without the application of electric or magnetic fields. (b) AES characterization of GO membrane after the penetration of KCl sources under the application of electric field (5 V, source to drain). (c) AES characterization of GO membrane after the penetration of KCl sources in the presence of magnetic field (50 mT, drain to source). The penetration experiments before AES measurements were conducted as illustrated in Figures 3a and c.

Table S1. Radius of ions.

Ion	Na^+	K^+	Mg^{2+}	Ca^{2+}	Cl^-
Radius of naked ion (Å)	0.95	1.33	0.65	0.99	1.81
Radius of hydrated ion (Å)	3.58	3.31	4.28	4.12	3.32

*These data are cited from the paper: Nightingale, E. R. Phenomenological theory of ion solvation. Effective radii of hydrated ions. *J. Phys. Chem.* **63**, 1381–1387 (1959).
